# Colistin heteroresistance in *Enterobacter* due to base heterozygosity at certain *phoP* and *phoQ* locations

**DOI:** 10.1128/aac.00713-25

**Published:** 2025-09-18

**Authors:** Chengcheng Wang, Yu Feng, Zhiyong Zong

**Affiliations:** 1 Center of Infectious Diseases, West China Hospital, Sichuan University 617661 , Chengdu, China; 2 Division of Infectious Diseases, State Key Laboratory of Biotherapy, Chengdu, China; 3 Laboratory of Pathogen Research, West China Hospital, Sichuan University 34753, Chengdu, China; 4 State Key Laboratory of Respiratory Health and Multimorbidity, Chengdu, China; University of Fribourg, Fribourg, Switzerland

**Keywords:** colistin heteroresistance, *Enterobacter*, *phoP*, *phoQ*, whole genome sequencing, base heterozygosity

## Abstract

Colistin heteroresistance (CHR) is a growing concern in clinical settings. We aimed to investigate CHR for its prevalence in *Enterobacter* strains, its impact on colistin treatment, and its genetic mechanisms. We analyzed 109 non-duplicated *Enterobacter* strains isolated from blood cultures. Minimum inhibitory concentrations (MICs) of colistin for each strain were determined using microdilution and CHR was assessed by population analysis profile (PAP) assays. *In vitro* time-killing assays and *in vivo* murine intra-abdominal infection models were conducted to evaluate whether CHR contributes to colistin treatment failure. Whole genome sequencing and single nucleotide polymorphism (SNP) analysis were performed to uncover the genetic mechanisms associated with CHR, which were verified using cloning experiments. About 30% of colistin-susceptible *Enterobacter* strains exhibited CHR, which indeed increased treatment failures. Novel base alterations in the two-component system gene *phoP* or *phoQ* were identified as the mechanism for colistin resistance. The presence of such colistin-resistance-mediated base alterations in minor subpopulations of the same strain was detected, which generates base heterozygosity resulting in heterogeneity of colistin resistance. Colistin resistance can be mediated by various mutations in the same strain after exposure to colistin, which provides the flexibility to accommodate antimicrobial selection pressure. In conclusion, these findings allow us to disclose the genetic heterogeneity in CHR *Enterobacter* strains, which is consistent with “phenotypic heterogeneity” and provides genetic explanations for the dynamic heterogeneous resistance. Our study underscores the clinical significance of CHR and provides important insights and new perspectives into the mechanisms for the heterogeneity of antimicrobial resistance.

## INTRODUCTION

*Enterobacter*, a genus of Gram-negative bacteria, is the third most common opportunistic pathogen within the family *Enterobacteriaceae* after *Escherichia* and *Klebsiella* (1). Colistin is a last-resort antimicrobial agent for treating carbapenem-resistant Gram-negative bacteria. However, colistin resistance has emerged globally, resulting in a concerning public health crisis that is exacerbated by the occurrence of colistin heteroresistance (CHR) (2, 3). CHR is a specific type of antimicrobial resistance in which certain colistin-resistant subpopulations of a bacterial strain lead to phenotypic heterogeneity of the entire population in susceptibility to colistin. The resistant subpopulations increase in size during exposure to colistin, but it remains controversial whether CHR leads to colistin treatment failure and a poor prognosis, as previous studies have reported inconsistent results (4, 5).

Heterogeneous resistance has two components: resistance and heterogeneity. However, mechanisms for the heterogeneity of CHR in Gram-negative bacteria remain largely unclear. Mechanisms for colistin resistance have been well characterized and can be due to chromosomal modifications or plasmid-borne *mcr* genes (1). In *Enterobacter*, colistin resistance is mainly mediated by the chromosomal PhoP-PhoQ two-component system (TCS) (6–11) and is also due to their regulators MgrB (12) and Ecr (9) and the global transcriptional system SoxS-SoxR (13), which regulates multiple genes to modify lipopolysaccharides (LPS). PhoP-PhoQ acts on the *arnBCADTEF* gene operon (6, 10, 14–16) and the AcrAB-TolC efflux pump (13, 14, 17). In addition, in several *Enterobacter* species, the TCS CrrA-CrrB (also called ECL_01761-ECL_01762 [18]) also mediates colistin via regulating the small membrane protein CrrC (17), which forms a complex with the efflux pump membrane protein KexD (17, 18). In contrast to non-heterogeneous colistin resistance mechanisms, those underlying heterogeneous colistin resistance are not fully understood. Allelic differences in *phoQ* and *mgrB* were found to determine the CHR level in different *Enterobacter* strains, but they failed to elucidate the mechanism mediating CHR in monoclonal strains (10). For monoclonal strains, variable expression of PhoP-PhoQ-regulated genes, in particular the *arnBCADTEF* gene operon (6, 9), has been assumed to contribute to the development of heterogeneity in colistin resistance. Tandem gene amplifications, nucleotide mutations, insertions, and deletions have also been proposed as important mechanisms contributing to colistin resistance heterogeneity in other *Enterobacteriaceae* (4). However, these multiple proposed mechanisms highlight the complexity of the formation of heterogeneous resistance and also suggest the likely presence of additional mechanisms, which are waiting for uncovering.

Here, we report that CHR is prevalent in *Enterobacter* clinical strains, and undetected resistant subpopulations in CHR strains may cause treatment failure and fatal infections based on *in vitro* and *in vivo* experiments. We found that base mutations in the *phoP* and *phoQ* genes were the mechanisms for colistin resistance. We elucidated the base heterozygosity, for which colistin resistance-mediated bases co-exist with those not associated with colistin resistance, at certain *phoP* and *phoQ* sites in the same *Enterobacter* CHR strain population. We provide evidence of “genetic heterogeneity” in *Enterobacter* strains as the genetic explanation for the dynamic nature of CHR, generating important insights into antimicrobial resistance mechanisms and clinical implications.

## RESULTS

### Colistin resistance was common in the 109 *Enterobacter* strains from blood

**Fig 1:**
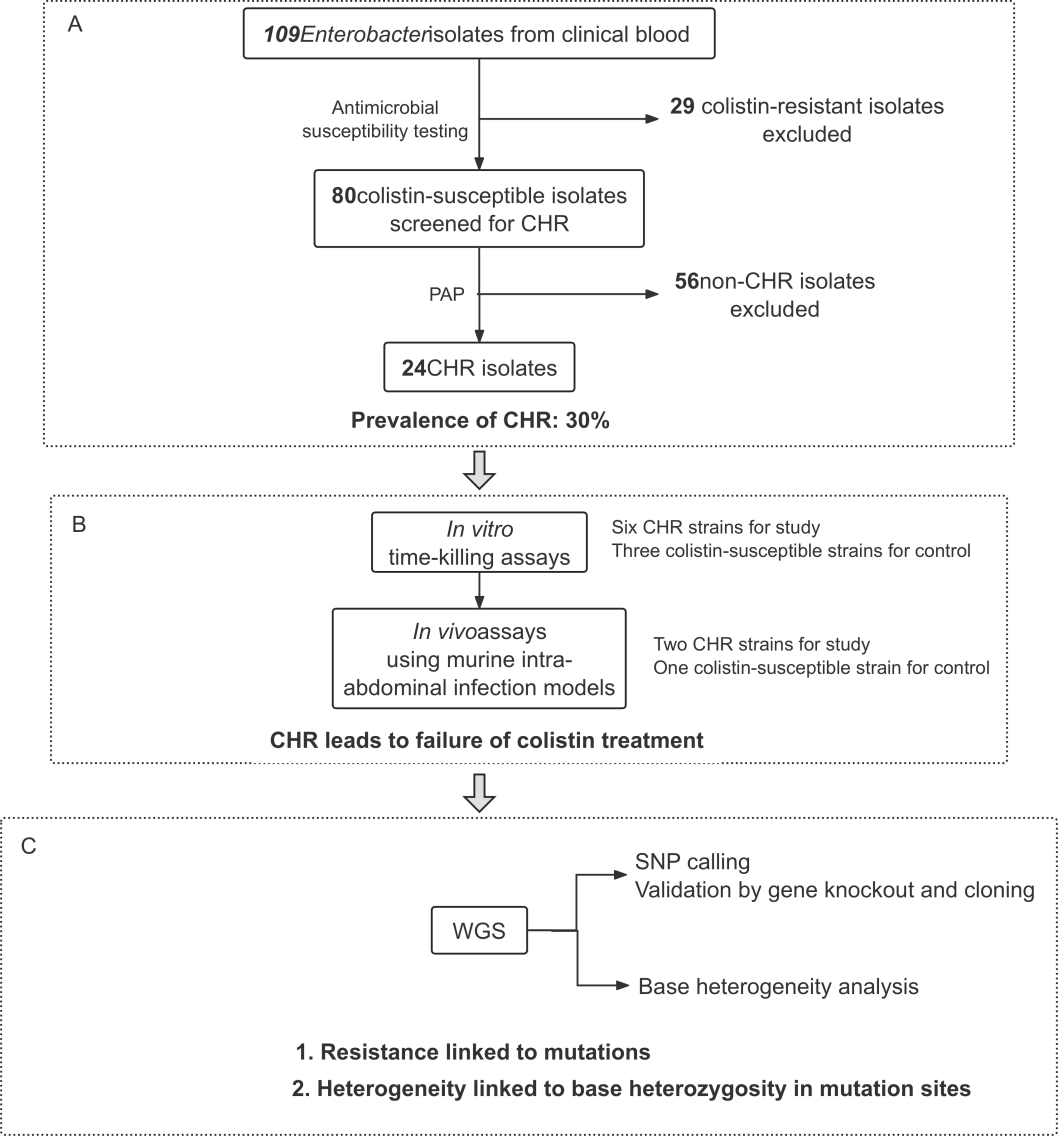
A schematic outline of methods and main results. (**A**) CLSI microdilution broth method was used to determine the MICs of colistin. PAP assays were performed to determine the presence of CHR. (**B**) To examine whether CHR led to treatment failure, we conducted *in vitro* time-killing experiments and *in vivo* assays using murine intra-abdominal infection models. (**C**) Mutations mediating colistin resistance were investigated by WGS for CHR strain 120027 and its derived isolates. The base heterozygosity at mutation sites was confirmed by WGS. MIC, minimum inhibitory concentration; PAP, population analysis profile; CHR, colistin heteroresistance; SNP, single nucleotide polymorphism; WGS, whole-genome sequencing.

During the 3.5-year study (January 2016–June 2019), 109 nonduplicated *Enterobacter* strains were recovered from blood cultures (Fig. 1; Data S1). Precise species identification based on *is*DDH and ANI uncovered *Enterobacter xiangfangensis* (47.7%, *n* = 52) and *Enterobacter hoffmannii* (22.0%, *n* = 24) as the most common species. The remaining 33 strains represented 13 other *Enterobacter* species (Data S1 for information of the 109 strains). These 109 strains were assigned to 73 sequence types (STs), including 29 novel STs, which are designated N1 to N29 here (Data S1). The most common STs were ST278 (*n* = 12; *E. hoffmannii*) and ST171 (*n* = 9; *E. xiangfangensis*). Of the 109 strains, 29 (26.6%) were colistin-resistant, with MICs ranging from 4 to 512 mg/L. Plasmid-borne *mcr* genes, either *mcr-9* (*n* = 6) or *mcr-10* (*n* = 2), were detected in three colistin-resistant and five colistin-susceptible strains (Data S1).

### Prevalence of CHR in *Enterobacter* strains

Of the 80 colistin-susceptible *Enterobacter* strains (73.4%), 30% (24/80) exhibited CHR, with most belonging to *E. xiangfangensis* (19/24, 79.2%; Data S1). The 24 CHR strains were assigned to various STs without discernible clustering (Table S1). Notably, the two most frequently identified STs in 109 *Enterobacter* strains, namely ST278 *E. hoffmannii* and ST171 *E. xiangfangensis,* rarely exhibited CHR. The 24 CHR strains displayed resistance/susceptibility (R/S) values of 16, 32, or 64 in the PAP assay and had a predominant colistin-susceptible population with a small colistin-resistant subpopulation able to grow on agar plates containing 4–16 mg/L colistin. The mutation frequencies of the 24 CHR isolates ranged from 1.2 × 10^−7^ to 4.7 × 10^−6^, with a median of 7.3 × 10^−7^ (Table S2). The majority of CHR isolates (18/24, 75%) exhibit a strong mutator phenotype, the remaining isolates (6/24, 25%) were weak mutators (>4.0 × 10^−8^ and ≤4.0 × 10^−7^) according to previously established criteria (19).

### CHR leads to failure of colistin treatment

**Fig 2:**
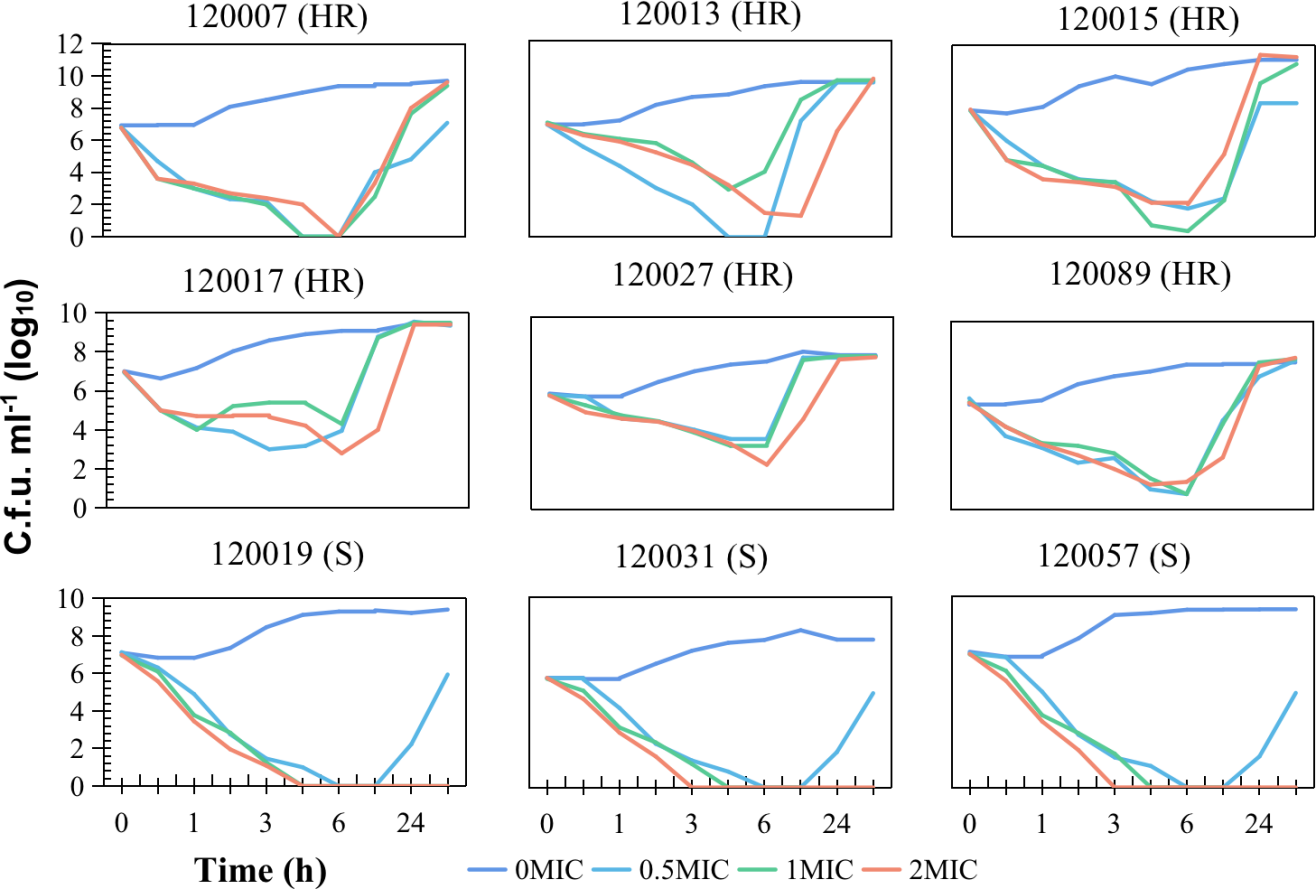
Time-killing experiments for *Enterobacter* strains. After administration of colistin at 1 mg/L (0.5× MIC), 2 mg/L (1× MIC), or 4 mg/L (2× MIC), the entire population of all strains with CHR (shown as HR in the figure) or without CHR (colistin-susceptible, indicated as S in the figure) decreased. However, the population of the six strains with CHR rebounded 4 or 6 h later, reaching the same level at 18 to 48 h compared with that without exposure to colistin. In contrast, rebounding of bacterial counts was only observed with exposure to colistin at 0.5× MIC but it did not reach the same level as without colistin exposure in the three colistin-susceptible strains.

**Fig 3:**
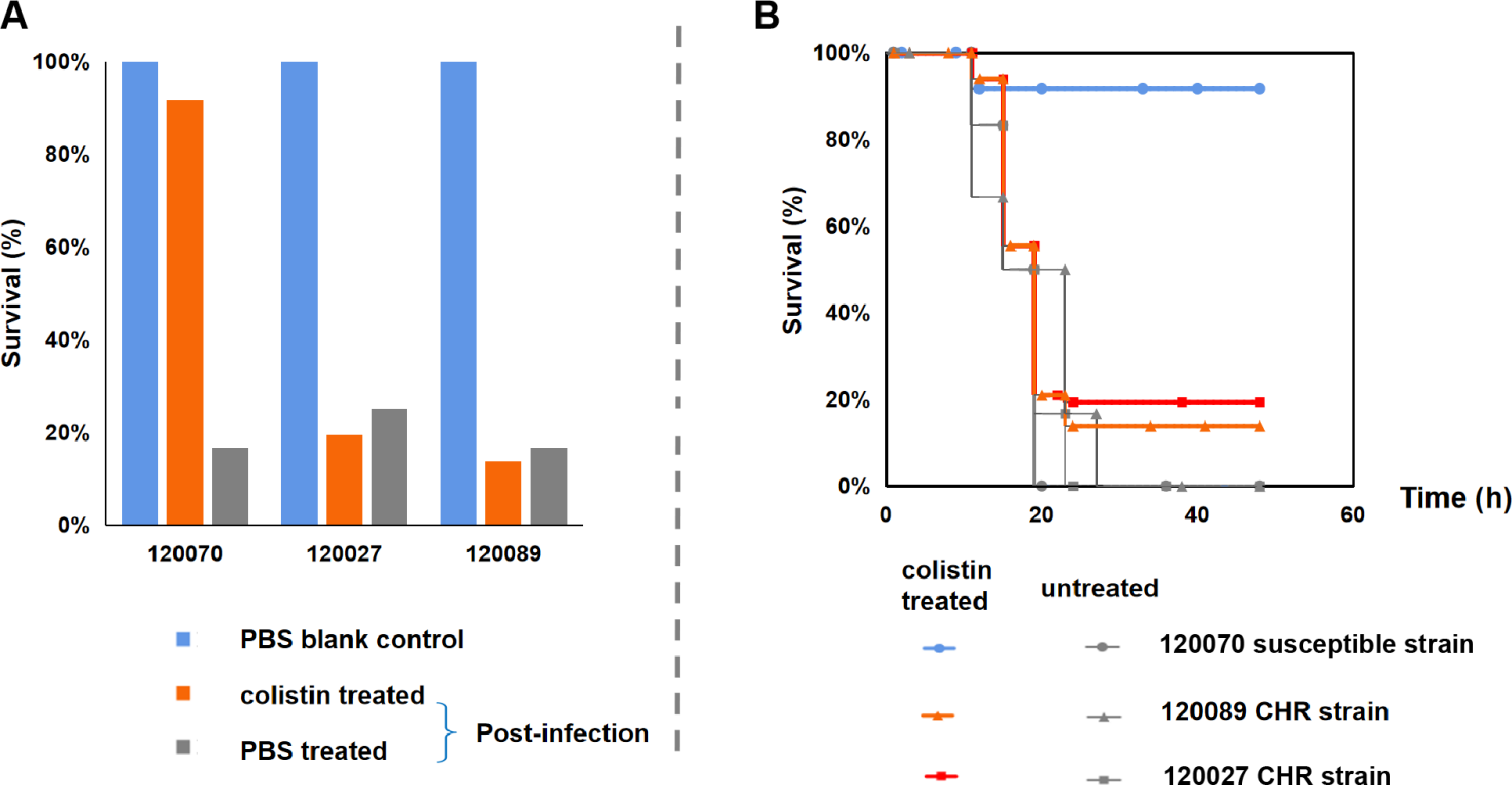
Murine intra-abdominal infection models for *Enterobacter* strains. (**A**) Survival rates of mice infected with two colistin - heteroresistant (CHR) strains (120027, 120089) and one colistin - susceptible strain (120070), comparing outcomes across PBS blank control, colistin - treated, and PBS - treated (post - infection) groups. (**B**) Survival curves of infected mice over time (up to 60 h), differentiating colistin - treated and untreated conditions for the three strains: 120070 (susceptible), 120089 (CHR), and 120027 (CHR). Mice were infected intraperitoneally with 0.1 mL of 3 × 10⁹ CFU/mL bacterial suspension, with colistin (10 mg/kg) or PBS administered every 6 h starting at 12 h post - infection.

CHR caused colistin treatment failure in both *in vitro* and *in vivo* models. In time-killing experiments, six CHR strains (*E. xiangfangensis* 120013, 120015, 120017, 120027, 120089; *E. mori* 120007) and three colistin-susceptible controls (*E. xiangfangensis* 120019, 120031; *E. hoffmannii* 120057) were examined. All nine strains had a colistin MIC of 2 mg/L. Six CHR strains showed initial bacterial decrease followed by a rebound after 6 h of colistin exposure at various concentrations (0.5×, 1×, or 2× MIC), reaching pre-treatment levels by 18 to 48 h (Fig. 2; Data S2). In contrast, colistin-susceptible strains only rebounded after 0.5× MIC colistin exposure, reaching a lower level than that without exposure to colistin. In murine intra-abdominal infection models, compared with the ineffective 5 and 15 mg/kg colistin doses, 10 mg/kg colistin showed significant differences in its efficacy against colistin-susceptible and CHR strains. Mice infected with CHR strains (120027 and 120089, *E. xiangfangensis*) exhibited low survival rates (19.4% and 13.9%, respectively) after 10 mg/kg colistin treatment, while 91.7% of mice infected with the colistin-susceptible strain 120070 (*E. xiangfangensis*) survived (Fig. 3; Tables S3). These results indicate that CHR leads to treatment failure.

### The existence of tiny resistance subpopulations within the main susceptible population generates heterogeneity

**Fig 4:**
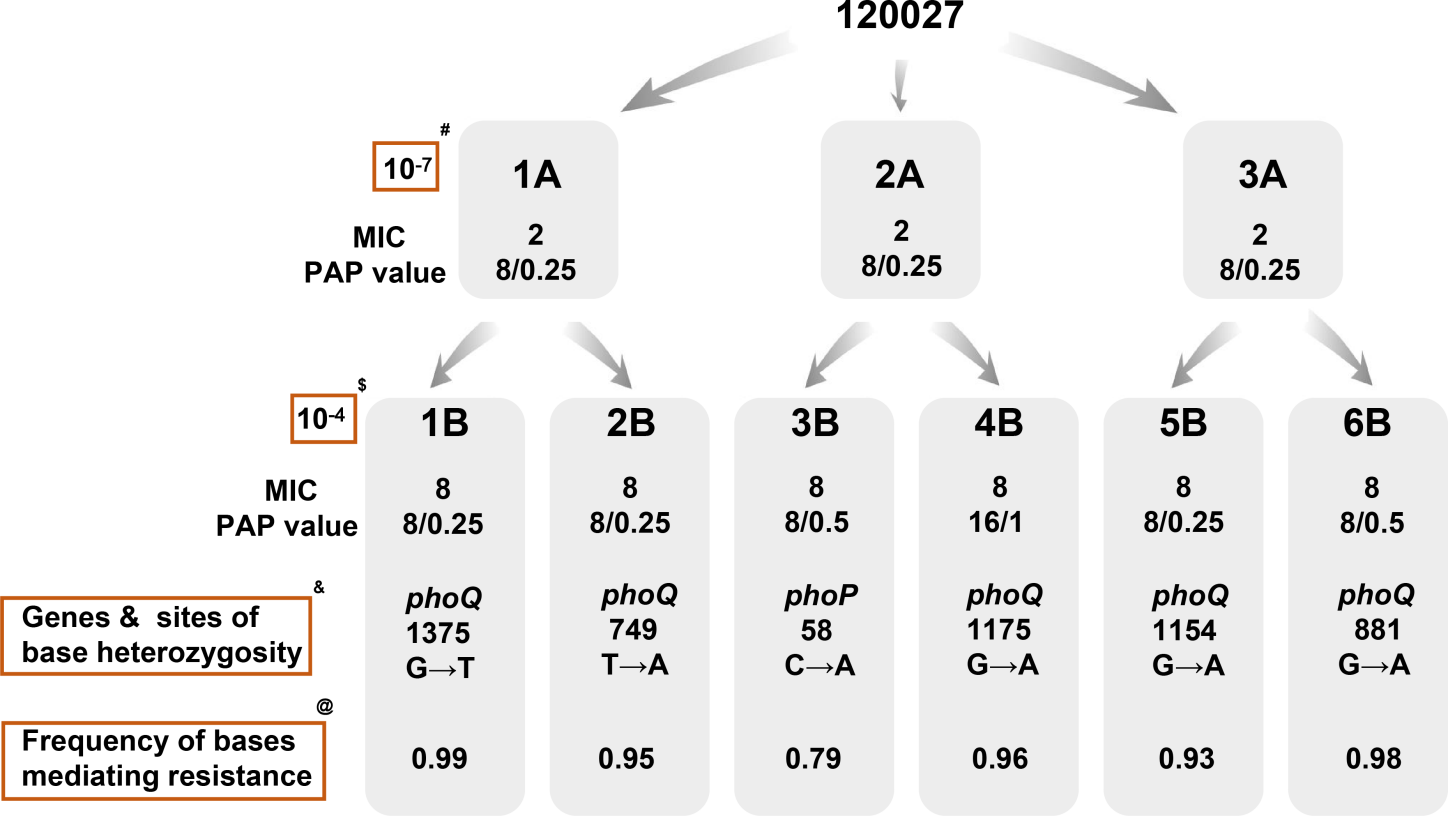
Phenotypic and genomic study scheme and results of a CHR representative strain. PAP value, the ratio of the colistin concentration at which the resistant subpopulation could grow (abbreviated as R) to the highest concentration at which the dominant susceptible subpopulation could grow (abbreviated as S). Three colonies of strain 120027 with CHR from a MH agar plate without colistin were randomly selected to represent three biological replicas and were assigned 120027_1A to _3A, respectively. All three isolates were susceptible to colistin (MIC, 2 mg/L) but exhibited CHR with an 8/0.25R/S ratio. Each of the three isolates was streaked onto a MH agar plate containing 8 mg/L colistin. After overnight incubation, for each of the three isolates, two colonies were collected from each agar plate to represent derived colistin-resistant isolates. Therefore, a total of six derived isolates were collected and assigned to 120027_1B to _6B. All six isolates were indeed resistant to colistin (MIC, 8 mg/L) and also exhibited CHR (R/S, 8/0.25 to 16/1). ^#^The frequency of resistant subpopulations was 3.34–6.67 × 10^−7^ in 120027_1A to _3A. ^$^The frequency of resistant subpopulations was 1.67–5.12 × 10^−4^ in 120027_1B to _6B. ^&^Genes and sites of base heterozygosity seen in 120027_1B to _6B. ^@^Frequency of bases mediating colistin resistance in 120027_1B to _6B (0.79 to 0.99), determined by whole-genome sequencing.

We selected a CHR strain 120027 (*E. xiangfangensis*) as a representative to study the mechanism of CHR (Table 1). Three biological replicas (120027_1A, 120027_2A, 120027_3A) from non-colistin plates had a colistin MIC of 2 mg/L (Fig. 4; Table S4). Despite being susceptible to colistin, all exhibited CHR with a 32R/S value (8/0.25 mg/L) in the PAP test, exceeding the 8R/S threshold for heterogeneous resistance. These replica isolates contained colistin-resistant subpopulations capable of growing on plates with 8 mg/L colistin, with a resistance proportion ranging from 3.34 × 10^−7^ to 6.67 × 10^−7^ as determined using PAP.

**TABLE 1:** Bacterial strains used for mechanism detection in this study^*a*^

Strain	Description or genotype
120027	Wild-type clinical CHR *E. xiangfangensis* strain
120027_1A	Replica 1 of 120027, CHR
120027_2A	Replica 2 of 120027, CHR
120027_3A	Replica 3 of 120027, CHR
120027_1B	Derived isolate of 120027_1A, replica 1, colistin-resistant
120027_2B	Derived isolate of 120027_1A, replica 2, colistin-resistant
120027_3B	Derived isolate of 120027_2A, replica 1, colistin-resistant
120027_4B	Derived isolate of 120027_2A, replica 2, colistin-resistant
120027_5B	Derived isolate of 120027_3A, replica 1, colistin-resistant
120027_6B	Derived isolate of 120027_3A, replica 2, colistin-resistant
25922	*Escherichia coli* ATCC 25922
120027_1A::pET-28	Tf of 120027_1A containing vector pET-28a alone
120027_1A::phoPQ^G1375T^	Tf of 120027_1A containing cloned *phoPQ*^G1375T^
120027_1A::phoPQ^T749A^	Tf of 120027_1A containing cloned *phoPQ*^T749A^
120027_1A::phoP^C58A^Q	Tf of 120027_1A containing cloned *phoP*^C58A^*Q*
120027_1A::phoPQ^G1175A^	Tf of 120027_1A containing cloned *phoPQ*^G1175A^
120027_1A::phoPQ^G1154A^	Tf of 120027_1A containing cloned *phoPQ*^G1154A^
120027_1A::phoPQ^G881A^	Tf of 120027_1A containing cloned *phoPQ*^G881A^
120027_1A_∆phoP	120027_1A with *phoP* gene knocked out
120027_1A_∆phoP::pET-28	Tf of 120027_1A_∆phoP containing pET-28a alone
120027_1A_∆phoP::phoPQ^G1375T^	Tf of 120027_1A_∆phoP containing cloned *phoPQ*^G1375T^
120027_1A_∆phoP::phoPQ^T749A^	Tf of 120027_1A_∆phoP containing cloned *phoPQ*^T749A^
120027_1A_∆phoP::phoP^C58AQ^	Tf of 120027_1A_∆phoP containing cloned *phoP*^C58A^*Q*
120027_1A_∆phoP::phoPQ^G1175A^	Tf of 120027_1A_∆phoP containing cloned *phoPQ*^G1175A^
120027_1A_∆phoP::phoPQ^G1154A^	Tf of 120027_1A_∆phoP containing cloned *phoPQ*^G1154A^
120027_1A_∆phoP::phoPQ^G881A^	Tf of 120027_1A_∆phoP containing cloned *phoPQ*^G881A^

^*a*^ Tf, transformant.

**Fig 5:**
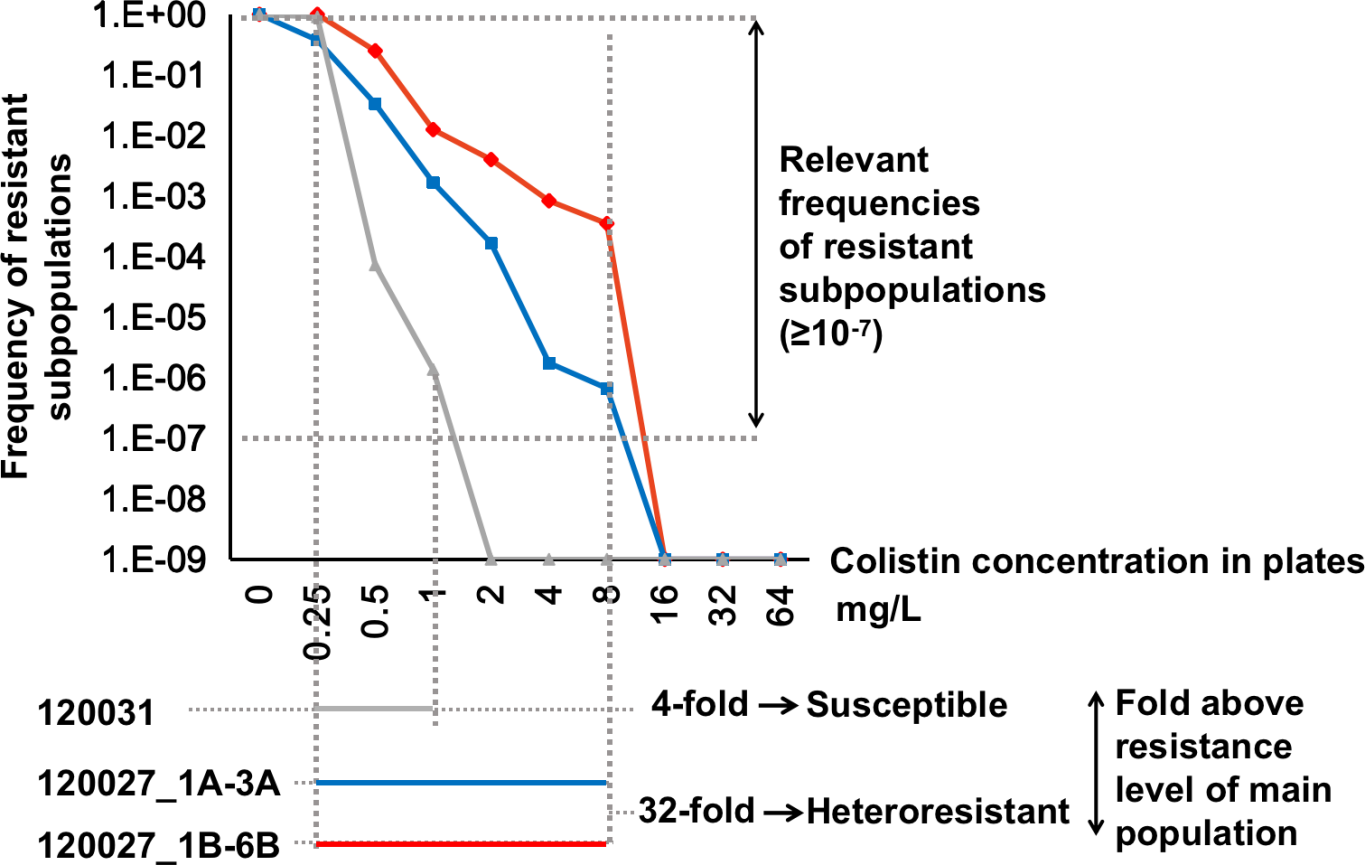
Frequency of resistant subpopulations in CHR strain 120027. The frequencies of resistant subpopulations in 120031, 120027_1A to _3A, and 120027_1B to _6B were 0, 3.34–6.67 × 10^−7^ and 1.67–5.12 × 10^−4^, respectively. 120031, a colistin-susceptible strain, was used as control. 120027_1A to _3A were three biological replicas of 120027, while 120027_1B to _6B were the derived colistin-resistant colonies of 120027_1A to _3A.

To enrich the resistant subpopulations, the replicas were cultured on 8 mg/L colistin plates, yielding six derived colonies (120027_1B to 120027_6B) (Fig. 4; Tables S4). These colonies were colistin-resistant (MIC 8 mg/L) and also exhibited CHR with a 16 or 32 R/S value (8/0.25, 8/0.5, or 16/1 mg/L), similar to their parental isolates. The proportion of resistant subpopulations in the six derived isolates increased to 1.67 × 10^−4^ to 5.12 × 10^−4^, about 1,000-fold higher than the 10^−7^ of their parental isolates (Fig. 4 and 5). This showed that exposure to colistin significantly increased the proportion of resistant subpopulations, demonstrating that a small number of resistant cells can replicate and drive the entire population toward colistin resistance.

### Nonsynonymous nucleotide mutations of *phoP* and *phoQ* mediate resistance to colistin

To determine the genetic mechanism behind CHR, all three replica isolates (120027_1A to 3A) and their six derived isolates (120027_1B to 6B) were subjected to whole genome sequencing (WGS) and single nucleotide polymorphisms (SNPs) calling. Genomic characteristics of these isolates were shown in Table S5. Compared with their parental isolates, five derived isolates had nonsynonymous mutations in *phoQ*, including G1375T (1B), T749A (2B), G1175A (4B), G1154A (5B), and G881A (6B), while 120027_3B had a mutation in *phoP* (C58A) (Fig. 4; Table S6). These mutations resulted in amino acid substitutions in PhoQ or PhoP.

**Fig 6:**
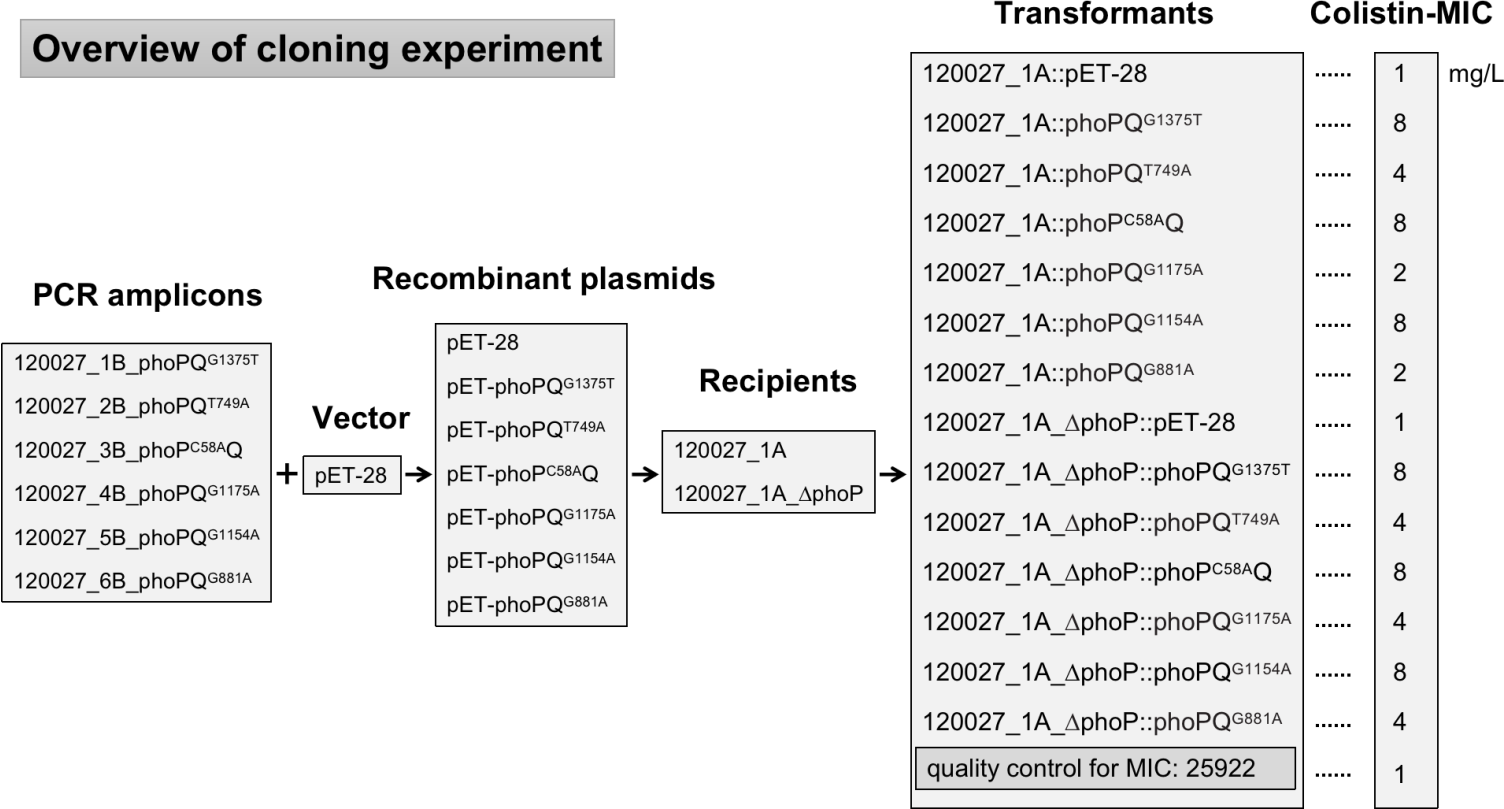
PCR amplicons, vectors, recipients, and transformants described in the cloning experiment and the colistin MIC of the transformants.

To confirm whether these nucleotide mutations in *phoP* and *phoQ* mediate colistin resistance, we cloned the mutated *phoP* and *phoQ* genes into pET-28a(+) (Miaolingbio, Wuhan, China) and transformed them into 120027_1A (Fig. 6). We therefore obtained transformants containing the original *phoP* and *phoQ* and the cloned mutated *phoP* and *phoQ*. Transformants with *phoQ*^G1375T^, *phoQ*^T749A^*, phoQ*^G1154A^, or *phoP*^C58A^ were resistant to colistin (MIC, four or 8 mg/L), confirming that these mutations could lead to colistin resistance. In contrast, transformants containing *phoQ*^G1175A^ or *phoQ*^G881A^ were susceptible to colistin, although the MIC of colistin against them was twofold (2 vs 1 mg/L) of that against the transformant containing the vector pET-28a alone. To eliminate the potential masking effect of the original *phoP* and *phoQ*, we knocked out *phoP* in 120027_1A using the CRISPR-Cas9 system, creating the strain 120027_1A_∆phoP. We then transformed recombinant plasmids into 120027_1A_∆phoP and found that colistin MIC increased to four or 8 mg/L in the presence of *phoQ*^G1175A^ and *phoQ*^G881A^, indicating these mutations also mediate colistin resistance (Fig. 6). The above findings also illustrate the coexistence of multiple resistance subpopulations containing different nucleotide mutations in a single strain. This highlights the heterogeneous, rather than homogenous, nature of cells in a single bacterial strain.

qRT-PCR analysis revealed upregulation of *phoP* and *arnA* in the resistant colonies compared with their parental colonies (Table 2). In addition, we observed no significant difference in the expression of the *tolC*, *soxS*, and *soxR* genes. This suggested the mutations in *phoP* and *phoQ* are associated with the lipopolysaccharide modification pathway, which has been known to contribute to colistin resistance (10).

**TABLE 2:** Gene expression levels in 120027_1B to _6B determined by qRT-PCR

Compared isolate	Reference	*phoP* ^*a*^	*arnA* ^*a*^	*tolC* ^*a*^	*soxS* ^*a*^	*soxR* ^*a*^
120027_1B	120027_1A	2.26	23.72	2.81	1.20	0.42
120027_2B	120027_1A	2.91	50.80	1.57	1.32	0.45
120027_3B	120027_2A	1.44	3.02	1.25	0.62	0.28
120027_4B	120027_2A	2.55	9.49	1.78	1.35	0.64
120027_5B	120027_3A	2.21	17.35	2.32	2.71	1.18
120027_6B	120027_3A	1.70	4.39	1.76	0.99	1.01

^*a*^ Genes that have been reported associated with colistin resistance in *Enterobacter*.

### Heterogeneity in resistance to colistin in *Enterobacter* stems from base heterozygosity and colistin selection

The existence of various colistin-resistant subpopulations together with the main colistin-susceptible subpopulations could result in heterogeneous resistance. Furthermore, we detected base heterozygosity in colistin-resistant 120027_1B to 6B at the previously detected *phoP* and *phoQ* mutant sites through whole genome sequencing (128× to 196× in depth) (Table 3). Under persistent colistin exposure, colistin resistance-mediated bases at specific *phoP* and *phoQ* sites accounted for 79% to 99% of all bases in colistin-resistant 120027_1B to 6B. However, no base heterozygosity was detected in their parental strains 120027_1A to 3A (Table 3). This may be the result of a low enough proportion of colistin-resistant subpopulations in the parental strains to be missed by standard WGS sequencing. The whole genome sequencing for 120027 was 128× to 196× in depth, which was only able to detect nucleotide base heterozygosity occurring at frequencies of 10^−3^ or above.

**TABLE 3:** Proportion of nucleotides (nt) mediating colistin resistance at the same position of *phoP* and *phoQ* in the parental isolates and their derived colistin-resistant isolates sequenced by WGS

Isolate	Gene	Position	At this position		
			Dominant nt in the parental population (no.)	Colistin-resistant nt (no.)	Proportion, colistin-resistant nt
120027_1A	*phoQ*	1375/1464	G (187)	T (0)	0
120027_1B	*phoQ*	1375/1464	G (2)	T (183)	0.99
120027_1A	*phoQ*	749/1464	T (170)	A (0)	0
120027_2B	*phoQ*	749/1464	T (6)	A (122)	0.95
120027_2A	*phoP*	58/675	C (186)	A (0)	0
120027_3B	*phoP*	58/675	C (35)	A (133)	0.79
120027_2A	*phoQ*	1175/1464	G (146)	A (0)	0
120027_4B	*phoQ*	1175/1464	G (7)	A (189)	0.96
120027_3A	*phoQ*	1154/1464	G (179)	A (0)	0
120027_5B	*phoQ*	1154/1464	G (13)	A (164)	0.93
120027_3A	*phoQ*	881/1464	G (165)	A (0)	0
120027_6B	*phoQ*	881/1464	G (2)	A (134)	0.98

## DISCUSSION

In this study, we report a high prevalence (30%) of CHR in colistin-susceptible *Enterobacter* strains in China. We then demonstrated that CHR indeed led to failure of colistin treatment using *in vitro* bactericidal assays and *in vivo* murine models. We identified that mutations at certain loci of *phoQ* and, to a lesser extent, mutations of *phoP* were the mechanism for colistin resistance in subpopulations of the same CHR strain. To the best of our knowledge, all mutations identified here have not been linked to colistin resistance before. We uncovered that a single locus of *phoP* and *phoQ* could contain various bases in the same strain, indicating base heterozygosity. We found that the proportion of different bases at the same locus remarkably changes in conditions with and without exposure to colistin, rendering flexibility to respond to stresses, such as selection pressure imposed by antimicrobial agents and driving the “heterogeneity” of antimicrobial resistance. We also observed the co-existence of multiple subpopulations, each of which contains a colistin resistance-mediated mutation, in the same strain. Taken together, we highlight the problem of CHR and elucidate the mechanism for both colistin resistance and its heterogeneity.

The high prevalence of CHR in this study echoes a 21.6% CHR prevalence among *Enterobacter* in the USA (2), indicating that CHR is common but often undetected in clinical settings, as heteroresistance is not routinely tested by clinical microbiology laboratories. *In vitro* and *in vivo* experiments uncovered that the bacterial load of CHR strains was not reduced by colistin treatment but increased by approximately 10-fold due to small colistin-resistant subpopulations, resulting in colistin treatment failure. This finding aligns with previous studies on colistin failure in CHR *Enterobacter, Klebsiella,* and *Acinetobacter* strains (8, 20, 21). Previous studies on *Enterobacter* CHR-related colistin treatment failure typically have focused on single *Enterobacter* strains (8). Our study adds to this by conducting *in vitro* time-killing assays on six different *Enterobacter* strains and *in vivo* murine infection models on two *Enterobacter* strains, confirming that CHR *Enterobacter* strains lead to colistin treatment failure. This further underscores the clinical significance of CHR, highlighting the need for careful monitoring when colistin is the agent of choice for bloodstream infections caused by *Enterobacter* strains.

Phenotypically, it appears that a limited number of resistant subpopulations have been able to proliferate in the presence of colistin, becoming dominant and mediating resistance. However, it is unclear from prior investigations what magnitude of these drug-resistant subpopulations might be for the entire population (4). Our results suggest that a proportional increase of at least 1,000-fold would be necessary to alter the MIC of the entire population from susceptible to resistant, necessitating more studies with a larger sample size and additional experimental validation. Despite the dominance of resistant subpopulations under the sustained action of colistin, certain susceptible ones persist in CHR strains, challenging the belief that colistin easily eradicates susceptible bacteria (22, 23). The mechanism underlying the coexistence and competition between resistant and susceptible subpopulations, particularly the persistence of susceptible subpopulations in the presence of colistin without being lysed, is currently unknown and requires further studies.

Importantly, we observed that colistin resistance in a single strain can arise from multiple colistin resistance-mediated mutations after colistin exposure. Under colistin pressure, any of the subpopulations with a colistin-resistance-mediated mutation could proliferate to protect the entire population from action of colistin. The presence of multiple colistin-resistant mutants could further diversify the availability of bet-hedging and therefore may amplify the flexibility of the host strain to accommodate stresses. Environmental factors and stochastic partitioning during cell division are thought to contribute to the occurrence of base heterozygosity in microcolonies (23, 24). The presence of base heterozygosity can lead to the evolution of important traits such as colistin resistance, ensuring the survival of individuals or the entire population in hostile environments. As such, the base heterozygosity has vital implications for understanding genetic diversity and colistin resistance in microbial populations.

It has been previously proposed that heteroresistance can be due to upregulation of the *arn* operon gene expression via the PhoPQ two-component system (10). In this study, we further demonstrated that heteroresistance consists of two distinct components, namely “resistance” and “heterogeneity.” While the upregulation of *arn* operon expression driven by *phoPQ* mutations explains the “resistance” mechanism, it fails to account for the “heterogeneity.” Compared with previous studies (9, 10), our study leads to a step further by identifying base heterozygosity at the gene level, providing a mechanistic explanation for 'heterogeneity' and advancing our understanding of heteroresistance mechanisms.

In conclusion, we discovered that CHR is common in *Enterobacter* and could cause treatment failure. We identified and verified new mutations in the two-component system *phoP* and *phoQ* as the mechanism for colistin resistance in small subpopulations within the entire population of a single strain. We uncovered base heterozygosity at certain loci of *phoP* and *phoQ* of a single strain and further illustrated the co-existence of multiple subpopulations with various colistin-resistance-mediated mutation in the same strain. We provide evidence for “genetic heterogeneity” in heteroresistance strains, which is consistent with “phenotypic heterogeneity” and genetically explains the unstable and transient resistance. This elucidates a novel mechanism and provides a new perspective for understanding heteroresistance to antimicrobial agents.

## MATERIALS AND METHODS

### Clinical strains and whole genome sequencing (WGS)

*Enterobacter* strains (*n* = 109) were recovered from clinical blood cultures at West China Hospital (Jan 2016–Jun 2019). Preliminary species identification was done using Vitek II (bioMérieux; Marcy-l’Étoile, France). Of these, 48 strains were previously sequenced (25); the remaining 61 were sequenced using the HiSeq X10 platform (Illumina; San Diego, CA, USA). DNA was extracted with the QIAamp DNA mini kit (Qiagen; Hilden, Germany) and DNA libraries prepared using the NEBNext Ultra II kit (NEB; Ipswich, MA, USA). Generated reads were assembled with Shovill v1.1.0 (https://github.com/tseemann/shovill) and quality checked using CheckM v1.0.18 (26). Genomes passing quality checks were annotated with Prokka v1.14.5 (27). Sequence types (STs) were determined using mlst v2.18.0 (https://github.com/tseemann/mlst). Antimicrobial resistance genes were identified via the ABRicate program (https://github.com/tseemann/abricate). Species identification was based on pairwise average nucleotide identity (ANI) and *in silico* DNA-DNA hybridization (*is*DDH) using JSpecies (jspecies.ribohost.com) (28) and GGDC (formula 2) (29), with 96% ANI (30) and 70% *is*DDH (29, 30) as cut-offs.

### Antimicrobial susceptibility testing

Minimum inhibitory concentrations (MICs) of colistin were determined using the microdilution broth method of the Clinical and Laboratory Standards Institute (CLSI) (31). A MIC >2 mg/L was considered resistance (CLSI and EUCAST, http://www.eucast.org/), while a MIC ≤2 mg/L was intermediate (CLSI) or susceptible (EUCAST). This study followed the EUCAST classification for colistin.

### PAP

PAP assays were conducted as described previously (4). Briefly, MH agar (Hopebio; Qingdao, China) plates containing no colistin or a varied colistin concentration of 0.125, 0.25, 0.5, 1, 2, 4, 8, 16, 32, and 64 mg/L were prepared. A single colony of each strain picked up from the MH agar plate containing no colistin was grown overnight in MH broth (Hopebio). Then, a 100-µL aliquot was streaked on a MH agar plate containing colistin at one of the 10 twofold serial concentrations from 0.125 to 64 mg/L and colonies were enumerated after overnight growth at 37°C. CHR was defined as a subpopulation of resistant cells that is capable of growing at a colistin concentration ≥8-fold higher than the highest concentration that the main susceptible population could grow at. We designated the “PAP value” to express the ratio of the colistin concentration at which the resistant population could grow (abbreviated as R) to the highest concentration at which the dominant susceptible population could grow (abbreviated as S). The PAP value is calculated as the ratio of R to S using the formula “PAP value = R/S.”

### Determination of mutation frequencies and classification of mutators

Mutation frequencies were assessed as described previously (32). Briefly, a single colony of each CHR isolate was resuspended in 20 mL of MH broth (Hopebio; Qingdao, China) and incubated overnight. Following centrifugation, the bacterial pellet was resuspended in 1 mL of sterile saline. Serial 10-fold dilutions of this suspension were then plated onto MH agar plates both with and without 4 mg/L colistin. After 48 h of incubation, colony counts were performed, and the proportion of colony-forming units (CFUs) on colistin-containing plates was calculated. All experiments were conducted in triplicate, with the mean value adopted as the mutation frequency for each isolate. If the variation among triplicate measurements exceeded a 10-fold range, the experiment was repeated. Isolates were categorized as strong mutators (i.e., mutation frequencies >4.0 × 10^−7^), weak mutators (i.e., mutation frequencies >4.0 × 10^−8^ and ≤4.0 × 10^−7^), or non-mutators (i.e., mutation frequencies ≤4.0 × 10^−8^) based on their mutation frequency as described previously (32).

### Time-killing experiments

Time-killing experiments were conducted as described previously (8) with minor modifications. Six CHR *Enterobacter* strains (*E. xiangfangensis*: 120013, 120015, 120017, 120027, 120089; *E. mori*: 120007) and three colistin-susceptible *Enterobacter* strains (*E. xiangfangensis*: 120019, 120031; *E. hoffmannii*: 120057) as control were tested. All nine strains had a colistin MIC of 2 mg/L. Overnight cultures (10^6^ CFU/mL) were inoculated into 5 mL MH broth with 0, 1, 2, or 4 mg/L colistin and incubated at 37°C for 48 h. At time points (0, 0.5, 1, 2, 4, 6, 8, 10, 24, and 48 h), bacterial suspensions were serially diluted and plated on MH agar without colistin. CFUs were counted after 24 h and used to plot a time-killing curve.

### Murine intra-abdominal infection models

Two CHR *Enterobacter* strains (120027, 120089; both *E. xiangfangensis*) and a colistin-susceptible strain (120070, *E. xiangfangensis*) were tested in female C57BL/6 mice (7 weeks, 20–22 g). Six mice per group were used to determine the minimum lethal dose (MLD), and 12 mice per group for treatment efficacy. The MLD was determined by increasing bacterial doses (from 5 × 10^8^, 1 × 10^9^, 3 × 10^9^, to 5 × 10^9^ CFU/mL), with 100% mortality occurring at 0.1 mL of 3 × 10^9^ CFU/mL (Table S7). Mice were inoculated intraperitoneally with 0.1 mL of 3 × 10^9^ CFU/mL bacterial suspension and treated with 5, 10, and 15 mg/kg colistin (or PBS for controls) 10-h post-infection, followed by every 6 h for 7 days. Survival was monitored every 6 h, with mice sacrificed at day 7.

### Obtaining derived isolates of 120027 and calling single nucleotide polymorphism (SNPs)

CHR strain 120027 (*E. xiangfangensis*) was selected for studying CHR mechanisms. Three biological replicas (120027_1A, 120027_2A, 120027_3A) were obtained from colonies on agar plates without colistin. These were then transferred to plates with 8 mg/L colistin to enrich resistant subpopulations. Six derived colonies (120027_1B to 120027_6B) were collected and sequenced for further analysis. SNPs were called by mapping the quality-controlled reads of derived isolates (120027_1B to 120027_6B) to their respective parental strains (120027_1A to 120027_3A) using Snippy v4.6.0 (https://github.com/tseemann/snippy).

### Knockout of the phoP gene and cloning experiment

The *phoP* gene of strain 120027_1A was knocked out using CRISPR-Cas9 gene editing technology (33). To assess if mutations in *phoP* and *phoQ* confer colistin resistance, the mutated genes *phoP* and *phoQ* were cloned into pET-28a(+) and transformed into 120027_1A to generate transformants with mutated phoP/Q. Detailed protocol is in the Supplementary Text.

### Quantitative reverse transcriptase PCR (qRT-PCR)

qRT-PCR was applied to measure the expression of *phoP*, *arnA*, *tolC*, *soxS*, and *soxR* genes in strains 120027_1B to _6B and 120027_1A to _3A. RNA was prepared using RNAiso Plus Kit (Takara) and cDNA was synthesized using PrimeScript RT Reagent Kit (Takara). qRT-PCR was performed using TB Green *Premix Ex Taq* II (Tli RNaseH Plus) kit (Takara) with self-designed primers (Table S8), and the housekeeping gene *rpoD* was used as the internal control (34).

### Identifying base heterozygosity

To identify the base heterozygosity in 120027_1B to _6B, the mapping and SNP calling were performed using Snippy v4.4.5 (https://github.com/tseemann/snippy), with sequence *phoQ* in 120027_1A used as references for 120027_1B and 120027_2B, *phoP* in 120027_2A for 120027_3B, *phoQ* in 120027_2A for 120027_4B, and *phoQ* in 120027_3A for 120027_5B and 120027_6B. If base heterozygosity was present, Snippy reported the base types and copy numbers at mutation sites. The alternative read rate was calculated from the number of alternative reads and total reads, with mapping depth information obtained from SAMtools (35).

## Data Availability

All relevant data are within the manuscript and its Supplemental material.

## SUPPLEMENTAL MATERIAL

Data S1 aac.00713-25-s0001.xlsx Clinical and genomic study of 109 Enterobacter strains in bloodstream infection, China.

Data S2 aac.00713-25-s0002.xlsx Time-killing experiments in Enterobacter isolates with CHR or susceptible to colistin.

Supplemental material aac.00713-25-s0003.docx Tables S1 to S8.

ASM does not own the copyrights to Supplemental Material that may be linked to, or accessed through, an article. The authors have granted ASM a non-exclusive, world-wide license to publish the Supplemental Material files. Please contact the corresponding author directly for reuse.
