# Supplementary material for: Colistin heteroresistance in Enterobacter due to base heterozygosity at certain phoP and phoQ locations
Source: Antimicrob Agents Chemother. 2025 Sep 18;69(11):e00713-25. doi: 10.1128/aac.00713-25 (PMC12587611; doi:10.1128/aac.00713-25)
Supplement: Supplemental material — Tables S1 to S8. [file aac.00713-25-s0003.docx]

**Supplementary Text**

**Detailed protocol for Knockout of the *phoP* gene of strain 120027_1A**

**A. Spacer cloning**

A 20-bp spacer sequence before a PAM site (5’-NGG-3’) targeting the *phoP* gene was picked using the CRISPR tool (http://crispr.dbcls.jp/). Spacer primers PhoP-Spacer-F (5′-TAGTTtagggctgccggatgaaga) and PhoP-Spacer-R (5′-AAACtcttcatccggcagccctaA) were synthesized in Sangon Biotech, Shanghai, China, and were phosphorylated using T4 Polynucleotide Kinase (New England Biolabs; Ipswich, MA, USA) and then annealed. The annealed oligos were ligated into the *Bsa*I-linearized sgRNA expression plasmid pSGKP-km (Addgene #117233) using T4 DNA ligase (New England Biolabs) to generate plasmid pSGKP-sgphoP. pSGKP-sgphoP was transformed into *E. coli* DH5α competent cells (Sangon Biotech) using the chemical method.(1) The successful cloning of pSGKP-sgphoP was verified by PCR using the primers phoP-spacer-F and M13R (5’-CAGGAAACAGCTATGACC) and by subsequent [Sanger sequencing](https://www.sciencedirect.com/topics/medicine-and-dentistry/sanger-sequencing).

**B. Preparation of single-stranded DNA repair template**

Two 50-nt DNA sequences flanking the *phoP* gene were spliced and then synthesized as a 100-nt single-stranded DNA (ssDNA) repair template named phoP_ssDNA (5’-cctgaataatcagcgttaaactattcataccatttataaagggagaagtgatgaaagggatattgcgccacattttacccctttcgctgcgggttcgctt).

**C. Preparation of competent cells and electroporation**

To prepare electrocompetent cells, 1 ml of overnight culture of *E. xiangfangensis*120027_1A was diluted 1:100 in 100 mL LB broth (Hopebio) at 37°C until the optical density at 600 nm (OD600) reached 0.5 to 0.7. The culture was then harvested by centrifugation at 5,000 × g for 10 min at 4°C. The supernatant was discarded, and the pellet was washed twice in ice-cold double distilled water (ddH_2_O) and sterile, ice-cold 10% glycerol, separately. Finally, the pellet was resuspended with 1 ml of ice-cold 10% glycerol and stored at -80°C to obtain 120027_1A-competent cells. The pCasKP-apr (Addgene #117231) plasmid was electroporated into 120027_1A-competent cells to obtain the 120027_1A-pCasKP strain. One milliliter of an overnight culture of 120027_1A-pCasKP was added to 100 mL of fresh LB broth supplemented with 30 mg/L apramycin (meilunbio, Dalian, China) and incubated at 30°C. One milliliter of 20% L-arabinose (meilunbio) was added into the LB broth when the OD600 of the culture reached 0.2. The pCasKP-harboring electrocompetent cells were prepared when the OD600 of the culture reached 0.5 to 0.7, as previously described.

**D. Genome editing**

The spacer-introduced pSGKP-sgphoP and phoP_ssDNA (donor repair template) were then co-electroporated into the L-arabinose-induced recipient cells harboring the pCasKP plasmid. The cells were plated onto a LB agar plate containing 30 mg/L apramycin (meilunbio) and 50 mg/L kanamycin (meilunbio), and the positive clones were screened by the primer pairs kn_phoP_up (5’-ccaggctcaggtcaacatact) and kn_phoP_down (5’-attccgcaggctcttactg) and verified by sanger sequencing. After curing both the pSGKP-km and pCasKP-apr plasmids, the resulting strain was named 120027_1A_ΔphoP.

**E. Plasmid curing**

For curing both the pCasKP-apr and pSGKP-km plasmids, a colony containing the desired *phoP* deletion was inoculated into antibiotic-free LB broth medium and incubated at 37°C for 12 h. Then, a fraction of the culture was streaked onto a LB agar plate containing 5% sucrose. The plate was incubated at 37°C for 12 h. Several colonies were streaked onto LB plates with or without the supplementation of apramycin or kanamycin, respectively, to confirm the successful curing. The colonies with the successful removal of both the pCasKP-apr and pSGKP-km plasmids could only grow on the plate without any antibiotics.

**Detailed protocol for cloning experiment**

The –10, –35 boxes within the promoter of phoP-Q were predicted using the online tool BPROM (<http://www.softberry.com/berry.phtml?topic=bprom&group>=programs&subgroup=g

fifindb). The complete coding sequence of phoPQ^G1375T^ in 120027_1B, phoPQ^T749A^ in 120027_2B, phoP^C58A^Q in 120027_3B, phoPQ^G1175A^ in 120027_4B, phoPQ^G1154A^ in 120027_5B, and phoPQ^G881A^ in 120027_6B, as well as their promoter regions were amplified with primers 120027_up_NotI (5′-AAAAAAGCGGCCGCCCAGGCTCAGGTCAACATACT; the restriction site is underlined) and 120027_down_XhoI (5′-AAAAAACTCGAGATTCCGCAGGCTCTTACTGA) using PrimeSTAR^®^ Max DNA Polymerase (Takara, Dalian, China). PCR amplicons and the vector pET-28a(+) (Miaolingbio, Wuhan, China) were digested using NotI and XhoI (NEB), respectively, and were ligated to the pET-28a(+) vector using T4 ligase (NEB) to construct pET-phoPQ^G1375T^, pET-phoPQ^T749A^, pET-phoP^C58A^Q, pET-phoPQ^G1175A^, pET-phoPQ^G1154A^, and pET-phoPQ^G881A^. Each of the above recombinant plasmids was separately transformed into colistin-susceptible strains 120027_1A and 120027_1A_∆phoP, respectively, by chemical transformation.(1) Potential transformants containing phoPQ^G1375T^, phoPQ^T749A^, phoP^C58A^Q, phoPQ^G1175A^, phoPQ^G1154A^, phoPQ^G881A^, respectively, were selected on LB agar plates (Hopebio) containing 50 mg/L kanamycin. The presence of phoPQ^G1375T^, phoPQ^T749A^, phoP^C58A^Q, phoPQ^G1175A^, phoPQ^G1154A^ or phoPQ^G881A^ in the corresponding transformant was verified by PCR using primers T7 (5′-TAATACGACTCACTATAGGG) and T7ter (5′-TGCTAGTTATTGCTCAGCGG) and subsequent [Sanger sequencing](https://www.sciencedirect.com/topics/medicine-and-dentistry/sanger-sequencing). MICs of colistin were determined for these transformants using the CLSI broth microdilution method.

**Table S1.** STs and species of the 24 colistin heterogeneous resistance (CHR) strains

| Species | STs | No. |
| --- | --- | --- |
| *E. ludwigii* | ST12 | 1 |
| *E. xiangfangensis* | ST50 | 3 |
| *E. xiangfangensis* | ST51 | 2 |
| *E. hoffmannii* | ST78 | 1 |
| *E. xiangfangensis* | ST113 | 1 |
| *E. xiangfangensis* | ST116 | 1 |
| *E. xiangfangensis* | ST175 | 1 |
| *E. xiangfangensis* | ST190 | 1 |
| *E. xiangfangensis* | ST306 | 1 |
| *E. roggenkampii* | ST422 | 1 |
| *E. xiangfangensis* | ST828 | 1 |
| *E. hoffmannii* | ST1126 | 1 |
| *E. xiangfangensis* | ST1160 | 1 |
| *E. xiangfangensis* | ST1248 | 1 |
| *E. mori* | STN2 | 1 |
| *E. xiangfangensis* | STN24 | 1 |
| *E. xiangfangensis* | STN4 | 1 |
| *E. xiangfangensis* | STN6 | 3 |
| *E. xiangfangensis*) | STN7 | 1 |

Table S2. Mutation frequencies and mutator classification of 24 CHR isolates

| Strain | Precise species | PAP value | Mutation frequencies | Mutator classification |
| --- | --- | --- | --- | --- |
| 120007 | *E. mori* | 4/0.125=32 | 6.1 × 10-7 | Strong mutators  (>4.0 × 10-7) |
| 120015 | *E. xiangfangensis* | 4/0.125=32 | 4.6 × 10-6 |  |
| 120017 | *E. xiangfangensis* | 8/0.5=16 | 3.8 × 10-6 |  |
| 120022 | *E. xiangfangensis* | 8/0.25=32 | 2.4 × 10-6 |  |
| 120027 | *E. xiangfangensis* | 8/0.25=32 | 1.1 × 10-6 |  |
| 120032 | *E. xiangfangensis* | 4/0.125=32 | 4.6 × 10-7 |  |
| 120033 | *E. roggenkampii* | 4/0.125=32 | 4.7 × 10-6 |  |
| 120036 | *E. xiangfangensis* | 8/0.25=32 | 4.5 × 10-6 |  |
| 120037 | *E. xiangfangensis* | 8/0.5=16 | 1.9 × 10-6 |  |
| 120054 | *E. xiangfangensis* | 8/0.5=16 | 6.2 × 10-7 |  |
| 120077 | *E. xiangfangensis* | 8/0.25=32 | 5.1 × 10-7 |  |
| 120089 | *E. xiangfangensis* | 8/0.25=32 | 7.7 × 10-7 |  |
| 120091 | *E. xiangfangensis* | 8/0.5=16 | 2.2 × 10-6 |  |
| 090004 | *E. xiangfangensis* | 16/0.5=32 | 6.0 × 10-7 |  |
| 090006 | *E. xiangfangensis* | 8/0.5=16 | 3.9 × 10-6 |  |
| 090017 | *E. ludwigii* | 8/0.25=32 | 4.3 × 10-6 |  |
| 090019 | *E. hoffmannii* | 16/0.5=32 | 6.9 × 10-7 |  |
| 090036 | *E. hoffmannii* | 4/0.25=16 | 9.9 × 10-7 |  |
| 120034 | *E. xiangfangensis* | 8/0.25=32 | 1.2 × 10-7 | Weak mutators  (>4.0 × 10-8 and ≤4.0 × 10-7) |
| 120087 | *E. xiangfangensis* | 8/0.125=64 | 3.6 × 10-7 |  |
| 090015 | *E. xiangfangensis* | 4/0.125=32 | 3.6 × 10-7 |  |
| 090026 | *E. xiangfangensis* | 4/0.125=32 | 3.3 × 10-7 |  |
| 090042 | *E. xiangfangensis* | 8/0.25=32 | 2.4 × 10-7 |  |
| 120013 | *E. xiangfangensis* | 4/0.25=16 | 3.0 × 10-7 |  |

Table S3. The mice infection-treatment survival experiment

| Groups | Strains | Minimum lethal dose of strains | Colistin dosage (mg/kg) | No. of survival | | | No. of overall survival |
| --- | --- | --- | --- | --- | --- | --- | --- |
|  |  |  |  | the 1^st^ time | the 2^nd^ time | The 3^rd^ time |  |
| colistin | - |  | 5/10/15 | 12 | 12 | 12 | 100.0% (36/36) |
| PBS | - |  | 0 | 12 | 12 | 12 | 100.0% (36/36) |
| PBS | 120070, colistin-susceptible | 3×10^9^  CFU/mL, 0.1 mL | 0 | 2 | 1 | 3 | 16.7% (6/36) |
| colistin |  |  | 5 | 5 | 4 | 4 | 36.1%(13/36) |
|  |  |  | **10** | **10** | **12** | **11** | **91.7% (33/36)** |
|  |  |  | 15 | 4 | 5 | 4 | 36.1%(13/36) |
| PBS | 120027,  CHR | 3×10^9^  CFU/mL, 0.1 mL | 0 | 3 | 3 | 3 | 25.0% (9/36) |
| colistin |  |  | 5 | 2 | 1 | 2 | 13.9%(5/36) |
|  |  |  | **10** | **2** | **2** | **3** | **19.4% (7/36)** |
|  |  |  | 15 | 3 | 2 | 1 | 16.7% (6/36) |
| PBS | 120089,  CHR | 3×10^9^  CFU/mL, 0.1 mL | 0 | 2 | 2 | 2 | 16.7% (6/36) |
| colistin |  |  | 5 | 3 | 2 | 1 | 16.7% (6/36) |
|  |  |  | **10** | **1** | **2** | **2** | **13.9% (5/36)** |
|  |  |  | 15 | 2 | 2 | 2 | 16.7% (6/36) |

PBS, phosphate buffered saline; CHR, colistin heteroresistance; CFU, colony forming unit.

10 mg/kg colistin showed significant differences in its efficacy against colistin-susceptible and CHR strains, and the results were in bold.

**Table S4.** MIC and population analysis profile (PAP) results of isolates derived from 120027

| Isolates | MIC  (mg/L) | PAP value | Proportion of resistant subpopulations | Isolates | MIC  (mg/L) | PAP value | Proportion of resistant subpopulations |
| --- | --- | --- | --- | --- | --- | --- | --- |
| 120027_1A | 2 | 8/0.25 | 6.67 × 10^-7^ | 120027_1B | 8 | 8/0.25 | 1.67 ×10^-4^ |
|  |  |  |  | 120027_2B | 8 | 8/0.25 | 3.27 ×10^-4^ |
| 120027_2A | 2 | 8/0.25 | 4.6 × 10^-7^ | 120027_3B | 8 | 8/0.5 | 2.67 ×10^-4^ |
|  |  |  |  | 120027_4B | 8 | 16/1 | 4.33 ×10^-4^ |
| 120027_3A | 2 | 8/0.25 | 3.34 × 10^-7^ | 120027_5B | 8 | 8/0.25 | 2.88 ×10^-4^ |
|  |  |  |  | 120027_6B | 8 | 8/0.5 | 5.12 ×10^-4^ |

Table S5. Genomic characteristics of 120027_1A to 3A and their derived isolates 1B to 6B

| colonies | Total length (bp) | Largest contig (bp) | N*50* | GC (%) | Contigs | contigs (>= 1000 bp) |
| --- | --- | --- | --- | --- | --- | --- |
| 120027_1A | 4745753 | 1008723 | 678542 | 55.52 | 36 | 16 |
| 120027_2A | 4749486 | 678738 | 456832 | 55.52 | 49 | 27 |
| 120027_3A | 4746823 | 1008741 | 429581 | 55.53 | 49 | 23 |
| 120027_1B | 4745776 | 1167698 | 725403 | 55.52 | 36 | 15 |
| 120027_2B | 4747419 | 1167922 | 725406 | 55.53 | 25 | 14 |
| 120027_3B | 4746026 | 1008741 | 479610 | 55.53 | 38 | 19 |
| 120027_4B | 4745695 | 1008435 | 690094 | 55.53 | 38 | 19 |
| 120027_5B | 4747409 | 1167922 | 725409 | 55.53 | 24 | 13 |
| 120027_6B | 4747346 | 1167922 | 725406 | 55.53 | 25 | 14 |

Table S6. SNPs of the six derived isolates compared to their parental isolates

| Query  isolate | Reference  isolate | Nucleotide  position^a^ | Genes | Amino acids  position^b^ | Protein |
| --- | --- | --- | --- | --- | --- |
| 120027_1B | 120027_1A | G1375T | *phoQ* | Gly459Cys | PhoQ |
| 120027_2B | 120027_1A | T749A | *phoQ* | Leu250Gln | PhoQ |
| 120027_3B | 120027_2A | C58A | *phoP* | Gln20Lys | PhoP |
| 120027_4B | 120027_2A | G1175A | *phoQ* | Cys392Tyr | PhoQ |
| 120027_5B | 120027_3A | G1154A | *phoQ* | Gly385Glu | PhoQ |
| 120027_6B | 120027_3A | G881A | *phoQ* | Arg294His | PhoQ |

^a^*phoP* and *phoQ* has 675 and 1464 nucleotides, respectively. The positions are numbered according to the start codon.

^b^PhoP and PhoQ has 224 and 487 amino acids, respectively.

Amino acids: Arg, arginine; His, hisosterine; Cys, cysteine; Tyr, tyrosine; Leu, leucine; Gln, glutamine; Lys, Lysine; Gly, Glycine; Glu, Glutamine.

SNPs, single nucleotide polymorphisms.

Table S7. The minimum lethal dose of bacterial strains

| Groups | Bacterial load, CFU/mL, 0.1 mL | strains | No. of survival (Time, post-infection) | | | | | | |
| --- | --- | --- | --- | --- | --- | --- | --- | --- | --- |
|  |  |  | 0 h | 10 h | 12 h | 16 h | 20 h | 24 h | 48 h |
| Load 1 | 5×10^9^ | 120070,  colistin-susceptible | 6 | 3 | 3 | 0 | 0 | 0 | 0 |
| Load 2 | 3×10^9^ |  | 6 | 5 | 5 | 3 | 0 | 0 | 0 |
| Load 3 | 1×10^9^ |  | 6 | 6 | 5 | 4 | 4 | 3 | 3 |
| Load 4 | 5×10^8^ |  | 6 | 6 | 5 | 5 | 5 | 4 | 4 |
| Load 1 | 5×10^9^ | 120027, CHR | 6 | 3 | 2 | 0 | 0 | 0 | 0 |
| Load 2 | 3×10^9^ |  | 6 | 6 | 5 | 3 | 1 | 0 | 0 |
| Load 3 | 1×10^9^ |  | 6 | 5 | 5 | 4 | 2 | 2 | 2 |
| Load 4 | 5×10^8^ |  | 6 | 6 | 6 | 5 | 4 | 4 | 4 |
| Load 1 | 5×10^9^ | 120089, CHR | 6 | 4 | 3 | 1 | 1 | 0 | 0 |
| Load 2 | 3×10^9^ |  | 6 | 5 | 4 | 3 | 2 | 1 | 0 |
| Load 3 | 1×10^9^ |  | 6 | 5 | 5 | 4 | 2 | 2 | 2 |
| Load 4 | 5×10^8^ |  | 6 | 6 | 6 | 5 | 3 | 3 | 3 |

Table S8. Primer sequence of qRT-PCR

| Primers’ name | Primer sequence 5′→3′ | Genes | Product length | TM (℃) |
| --- | --- | --- | --- | --- |
| phoP-up | CAGGAGATGGGTCATCAGGT | *phoP* | 158 bp | 60.5 |
| phoP-dw | AGGGAAACATCATGGCTACG | *phoP* |  | 58.4 |
| arnA-up | GTTGAAGGGGACATCAGCAT | *arnA* | 187 bp | 58.4 |
| arnA-dw | TGCGCTTATCGTATTTCACG | *arnA* |  | 56.4 |
| soxS_up | TCAAACGCTGGGTGATTACA | *soxS* | 185 bp | 56.4 |
| soxS_dw | AATTGAGCTGATGGCGGTAG | *soxS* |  | 58.4 |
| soxR_up | GGTGAAGTGGCTAAGCGAAG | *soxR* | 167 bp | 60.5 |
| soxR_dw | AGAGGAATGCCGATACGTTG | *soxR* |  | 58.4 |
| tolC_up | GTCTGGGCAACCCTGATTTA | *tolC* | 203 bp | 58.4 |
| tolC_dw | CATGTCGAACAGGGTTTGTG | *tolC* |  | 58.4 |

**References**

1. Sambrook J, Russel DW. 2001. Molecular cloning: a laboratory manual. Cold Spring Harbour, NY: Cold Spring Harbour Laboratory Press 1:3rd edition.
